# Early‐Life Host–Microbial Interactions and Asthma Development: A Lifelong Impact?

**DOI:** 10.1111/imr.70019

**Published:** 2025-03-18

**Authors:** Céline Pattaroni, Benjamin J. Marsland, Nicola L. Harris

**Affiliations:** ^1^ Department of Immunology, School of Translational Medicine Monash University Melbourne Victoria Australia

**Keywords:** asthma, early life, microbiota

## Abstract

Childhood is a multifactorial disease, and recent research highlights the influence of early‐life microbial communities in shaping disease risk. This review explores the roles of the gut and respiratory microbiota in asthma development, emphasizing the importance of early microbial exposure. The gut microbiota has been particularly well studied, with certain taxa like *Faecalibacterium* and *Bifidobacterium* linked to asthma protection, whereas short‐chain fatty acids produced by gut microbes support immune tolerance through the gut–lung axis. In contrast, the respiratory microbiota, though low in biomass, shows consistent associations between early bacterial colonization by *Streptococcus*, *Moraxella*, and *Haemophilus* and increased asthma risk. The review also addresses the emerging roles of the skin microbiota and environmental fungi in asthma, though findings remain inconsistent. Timing is a critical factor, with early‐life disruptions, such as antibiotic use, potentially leading to increased asthma risk. Despite significant advances, there are still unresolved questions about the long‐term consequences of early microbial perturbations, particularly regarding whether microbial dysbiosis is a cause or consequence of asthma. This review integrates current findings, highlighting the need for deeper investigation into cross‐organ interactions and early microbial exposures to understand childhood asthma pathophysiology.

## 1 Introduction

Childhood asthma poses a significant public health challenge, affecting more than 12% of children worldwide, with the highest rates seen in high‐income countries [1]. If not effectively managed, childhood asthma can lead to diminished lung function and severe respiratory complications as children transition into adulthood [2], elevating the risk of early‐onset comorbidities and even premature mortality [3]. Although genetic predisposition plays a critical role, it is now evident that environmental factors, especially microbial exposures in early life, can have a profound impact on asthma development [4].

From birth, our bodies are colonized by a diverse array of microbes that interact with the immune system and play a critical role in its development. These microbial communities—on the skin, in the gut, and in the respiratory tract—are established very early in life and help train the developing immune system. The “hygiene hypothesis” suggests that reduced exposure to microbes, especially in industrialized environments with increased sanitation, has contributed to the rising rates of allergic diseases, including asthma [5]. Central to this hypothesis is extensive research, both from mechanistic animal studies and large human cohort analyses, showing that early microbial exposure is essential for proper immune development, and disruptions during this critical window can have long‐term consequences. For example, early colonization of the respiratory tract by bacteria such as *Moraxella* and *Haemophilus* has been linked to persistent wheezing and asthma development. Similarly, disruptions in gut microbial diversity, particularly reductions in beneficial bacteria like *Bifidobacterium* and *Faecalibacterium*, are associated with increased asthma susceptibility. Importantly, the timing of these microbial interactions is critical, with the first 100 days of life representing a key window for immune programming [6].

Environmental exposures also play a crucial role in shaping asthma risk. The well‐documented “farm effect” demonstrates that children exposed to diverse microbial environments, such as farms, are significantly less likely to develop asthma [7]. Mechanistically, the gut–lung axis, particularly through the action of short‐chain fatty acids (SCFAs), has been the most thoroughly investigated [8]. Moreover, research suggests that the interactions between other organs, such as the skin and the nervous system through axes like the skin–lung [9] and gut–neuronal‐lung axes [10], may further influence asthma risk. Additionally, the role of fungi in asthma development is receiving increased attention [11], but the evidence remains inconsistent.

In this review, we will explore how early microbial influences, both intrinsic and extrinsic, shape immune development in ways that either increase or reduce asthma risk, with a primary focus on human studies to provide a comprehensive insight into the role of microbiota in childhood asthma.

## 2 The Foundations of Microbial Influence on Immune System Development

### 2.1 Seeding of the Microbiota: From “Sterile” Womb to Microbial‐Rich Environment

The transition from womb to world marks life's most dramatic environmental shift, as the newborn emerges from the protective amniotic sac and experiences initial exposure to a vast array of environmental variables including a plethora of microorganisms. According to the *sterile womb paradigm* [12], newborns acquire microbes both vertically from their mothers and horizontally from other humans or the environment during and after birth. The advent of next‐generation sequencing techniques, which offer enhanced sensitivity for microbiome investigations, has enabled studies that challenge this paradigm [13, 14, 15, 16]. A decade ago, Aagaard et al. [14] conducted the first large‐scale study describing a placental microbiota using both 16S amplicons sequencing and shotgun metagenomics. However, numerous subsequent studies have provided substantial evidence that these bacterial signals can be attributed to processing contamination [17] or contamination during sampling or birth itself [18]. As highlighted in *Nature*'s opinion review in 2023 [19], the current consensus in the field refutes the idea of in utero microbiota acquisition [20, 21].

The initial microbiota of a newborn is largely undifferentiated across body sites [22, 23] and rapidly evolves to become tissue‐specific as the infant develops. Multiple studies have demonstrated that the mode of delivery significantly impacts an infant's initial microbial colonization [22, 23, 24], with vaginal births facilitating the transfer of microbes from the mother's reproductive tract and intestinal microbiota, whereas caesarean deliveries result in neonatal microbiomes predominantly populated by bacteria seeded from the maternal skin flora [22, 23]. A recent study revealed that approximately 60% of an infant's microbiota originates from maternal sources, with C‐section births altering transmission patterns by reducing fecal microbiota transfer but increasing breast milk microbiota colonization [25].

In the airways, differences between vaginally and C‐section delivered infants are mainly observed around birth [23], with the following months showing minimal differences, except for slightly delayed colonization patterns and greater microbial instability in C‐section infants, who experience more frequent shifts in the types of bacteria present and have lower abundances of beneficial bacteria like *Corynebacterium* and *Dolosigranulum* during the first 6 months of life [26]. Interestingly, in the lower airways of newborns during the first week of life, differences in microbial composition of vaginally or C‐section born children were only observed in preterm babies [27], possibly reflecting the less developed immune system and airways of preterm infants. As Caesarean‐born infants have a 20% increase in asthma risk [28, 29, 30], it is important to investigate the impact of C‐section delivery on early microbial colonization in the gut and respiratory tract, as disruptions in microbial seeding may potentially be linked to the elevated asthma risk observed in these infants.

### 2.2 Lung Immune Responses: Rapid Evolution After the First Breath

The relatively limited exposure to antigens in utero does mean that newborns are more reliant on innate immune pathways for protection against infections immediately after birth. Although infants often experience more severe outcomes from certain infections compared to adults [31, 32], this observation does not necessarily indicate the lack of an effective immune system but may instead reflect a different state of maturation or activation. Indeed, the human fetal immune system proves to be more complex than initially believed, comprising all cellular components of both innate and adaptive immunity. T cells start populating the peripheral tissues by 12–14 weeks of human gestation [33, 34] and rapidly proliferate in response to homeostatic signals, driven by a combination of maximal thymopoiesis and high proliferative expansion of mature naive T cells [35]. The activation and proliferation of immune cells are meticulously controlled and inhibited by regulatory T cells (Tregs) [36, 37], whose suppressive activity decreases during human term labor [38]. A recent study [39] investigating human embryonic and fetal lung immune cells revealed a biphasic pattern where progenitor and innate immune cells dominate from the embryonic stage and early pseudoglandular stage before being gradually replaced by T‐ and B‐lymphocytes. Notably, the research highlighted that B‐cell maturation occurs in the lungs—challenging the belief that the bone marrow is the sole source of mature B cells—and showed that lung mesenchyme and epithelium support B‐cell homeostasis through the secretion of chemokines.

At birth, the transition from the sterile environment of the womb to the outside world exposes the alveoli to air and microbes for the first time. This sudden exposure, combined with the mechanical forces of the spontaneous initial ventilation [40], triggers significant immune events. Two independent murine studies [41, 42] have reported the sudden release of IL‐33 by alveolar type II (AT2) cells at birth. This IL‐33‐driven process leads to a wave of Type 2 immune cell expansion and activation, particularly affecting innate lymphoid cell type 2 (ILC2s) and eosinophils, and shapes the functional performance of newly differentiating alveolar macrophages and dendritic cells (DCs). However, it is important to remember that human and mouse lungs differ significantly at birth, with human lungs being more structurally developed and the immune system more mature, featuring a higher diversity of immune cells. In humans, IL‐33 was found to be highly expressed in the lower airways of term‐born infants with diverse microbiota but was absent in preterm infants during their first week of life [27]. This early‐life Th2 response in the lung, characterized by IL‐33 and ILC2 activation, is a normal part of immune development and necessary for initial lung homeostasis. However, while essential initially, its overactivation can lead to asthma. Therefore, the subsequent modulation of this immune response is critical for long‐term respiratory health, highlighting the importance of tightly regulated immune responses in early life.

Another crucial aspect of the swift immune adaptation process, as the newborn's immune system adjusts to external environmental exposures, is the establishment of immune tolerance, with Tregs playing a pivotal role. In mice, exposure to house dust mite (HDM) antigens within the first 2 weeks of life—but not beyond this window—has been shown to confer protection against allergic airway inflammation [43]. This protection is mediated by the emergence of a specific subset of Helios‐negative Tregs. Notably, the induction of these Tregs is contingent upon microbial exposure, with a concomitant increase in bacterial load, particularly of the Bacteroidetes phylum, in the lungs. Similar to the lung, another study demonstrated that Tregs accumulate in the skin of neonatal mice in response to colonization by skin commensals during a critical 2‐week period after birth [44]. The gut microbiota also plays a central role in immune tolerance by producing SCFAs, which can enhance the suppressive activity of Tregs [45, 46, 47]. In vitro studies demonstrated that fecal water extracts from human neonates with a microbial profile associated with high asthma risk impaired the suppressive function of Tregs [48]. More recently, early‐life serotonin production by the gut microbiota has been shown to specifically enhance the expansion and stability of peripheral Tregs via serotonin receptor signaling, contributing to immune tolerance [10].

### 2.3 Microbial Players: Components and Dynamics of the Microbiota

To understand the dynamics of the microbiota and its potential dysregulation, it is important to recognize that its composition is intricately shaped by the specific microenvironments at various body locations. For instance, within the respiratory tract, distinct microbial communities are found in different regions: the nasal cavity and nasopharynx are dominated by *Moraxella*, *Staphylococcus*, *Corynebacterium*, *Haemophilus*, and *Streptococcus* species, whereas the oropharynx is rich in *Prevotella*, *Veillonella*, *Streptococcus*, *Leptotrichia*, *Rothia*, *Neisseria*, and *Haemophilus* species [49]. This spatial variation is similarly observed in the gut, although comprehensive human data is limited due to sampling challenges. Significant differences exist between microbial communities in stool samples and those adherent to the mucosal surfaces of the colon, duodenum, stomach, and mouth [50, 51]. For example, Firmicutes are more prevalent in stool samples, whereas *Faecalibacterium* and uncultured TM7 phylum bacteria are consistently found across various sites and individuals [51]. The skin also exhibits location‐driven microbial diversity: sebaceous regions such as the head, neck, and upper torso favor lipophilic microorganisms such as *Cutibacterium*, *Staphylococcus*, and *Malassezia* fungi, whereas moist areassuch as the armpits and groin support a broader range of species, predominantly *Corynebacterium* and *Staphylococcus* [52].

When examining the lung microbiota, it is crucial to understand its unique characteristics compared to the gut or skin. The lungs must maintain a low bacterial load to ensure efficient gas exchange, relying on mechanisms such as mucociliary clearance and coughing to expel microbes that reach the lower airways. Unlike the relatively stable microbiomes of the oral cavity and gastrointestinal tract, the lower airway microbiome is thought to be in constant flux due to episodic microbial exposure and rapid clearance [53]. Despite the transient presence of these microbes, they can leave lasting imprints on the immune tone, the baseline state of immune readiness and balance between activation and suppression of the lower airways. For instance, in mice, the aspiration of oral commensals, such as *Prevotella melaninogenica* , *Veillonella parvula* , and *Streptococcus mitis* , activates CD4+ and CD8+ T cells, recruits IL‐17‐producing T cells, and triggers counter‐regulatory immune responses, such as increased Tregs and immune‐checkpoint inhibitor markers on T cells [54].

An important concept in the field is microbial dysbiosis, defined as a deviation from normal microbial composition, involving changes either in the relative abundance or the diversity of microbes. As will be detailed in Sections 3.2 and 3.3, dysbiosis in the respiratory and gastrointestinal tracts has been linked to the development of childhood asthma, but it is unclear whether it is a cause or a consequence of the disease. Dysbiosis may precede asthma by altering immune responses, as suggested by studies showing that early colonization of the airways with bacteria like *Moraxella*, *Haemophilus*, and *Streptococcus* is linked with wheezing and asthma development years later [55, 56, 57, 58]. Conversely, asthma‐related changes in lung architecture and impaired clearance mechanisms could also lead to dysbiosis. Additionally, dysbiosis can both precede [57, 59] and follow [60] viral infections, suggesting a bidirectional relationship where it can either set the stage for infections or result from them, which could further complicate the asthma development process.

### 2.4 Microbial Milestones: Development of the Microbiota in Early Life

Early life is characterized by developmental milestones that occur within specific timeframes. Likewise, the lung and gut microbiotas develop through a sequence of microorganism acquisition, colonization, and selection, starting at birth and continuing through the first months/years of life until they achieve an adult‐like state, with differences influenced by the mucosal environment and external factors. Longitudinal studies have revealed that microbial colonization of the upper airways occurs very rapidly within the first week of life [26, 60]. Similarly, in the lower airways, a diverse microbiota is present within the first days of life, with the microbial community reaching a stable diversity by approximately 2 months of age [27]. Bacterial colonization of the airways starts with a high relative abundance of *Staphylococcus* spp., followed by the enrichment of *Corynebacterium* and *Dolosigranulum* spp. in the upper airways and *Streptococcus*, *Neisseria*, and *Prevotella* in the lower airways [26, 27, 60]. *Moraxella*, a pathobiont associated with asthma that will be further discussed in Section 3.2, also appears in the first weeks of life. Interestingly, this niche‐specific differentiation additionally occurs for fungi within the first week of life [61].

**FIGURE 1:**
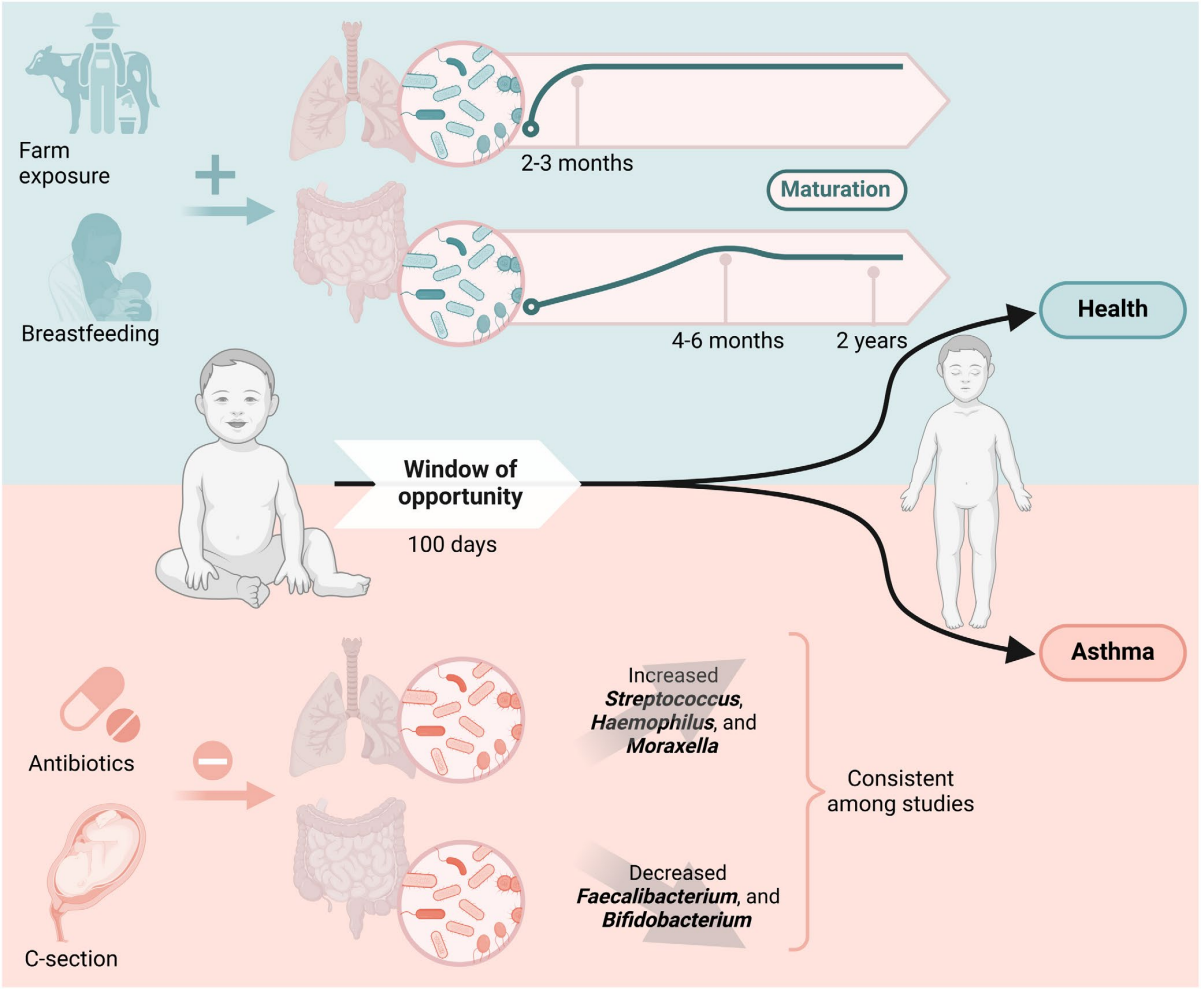
Early‐life microbiota and childhood asthma. The top half (green background) represents protective factors such as farm exposure and breastfeeding. The maturation of the airway microbiota occurs rapidly within the first 2–3 months of life, whereas the gut microbiota evolves more slowly, influenced by factors such as diet and the cessation of breastmilk. The gut microbiota does not fully stabilize until around 2–5 years of age. The bottom half (orange background) depicts disruptions to this process, such as antibiotic use and C‐section delivery, which can alter microbial colonization. Increased abundance of respiratory pathogens like *Streptococcus*, *Haemophilus*, and *Moraxella* and decreased levels of beneficial gut bacteria like *Faecalibacterium* and *Bifidobacterium* have been consistently linked to higher asthma risk. The figure emphasizes the 100‐day “window of opportunity” during which microbial exposures have the most significant impact on immune programming and asthma outcomes. Depending on these early‐life influences, children may follow a path toward either health or asthma development.

In contrast to the microbiota of the respiratory airways, which stabilizes quickly, the gut microbiota in infants experiences significant changes during the early years of life, reaching a composition similar to that of an adult between the ages of 2 and 5 (Figure 1) [62, 63, 64, 65]. The initial microbial composition of a newborn's meconium is characterized by low species richness and is predominantly made up of bacteria from the Firmicutes phylum, including genera such as *Enterococcus*, *Staphylococcus*, *Streptococcus*, and *Lactobacillus* [23, 62, 66, 67]. These bacteria are commonly found in other body sites of the newborn at birth, such as the oral cavity, skin, and nares, suggesting a shared initial colonization or “seeding” process, with only a few bacteria, such as *Escherichia* and *Klebsiella*, being specific to the gut [23]. When examining the relationship between gut microbiota and childhood asthma, it is crucial to consider the impact of early life diet on microbial and immune development. Breastfeeding represents an important microbial milestone, providing essential nutrients and bioactive components such as human milk oligosaccharides (HMOs) and IgA. HMOs support the growth of beneficial microbes such as *Bifidobacterium infantis* [68, 69], whereas IgA supports the colonization of specific microbes by enhancing their attachment to the intestinal lining [70]. The transition from breastfeeding to solid foods around 4–6 months marks the most significant turning point, leading to an increase in *Bacteroides* and *Clostridiales* species and a decrease in *Bifidobacteria* [62, 71, 72], directly linked to the cessation of breastfeeding [73]. This dietary change, along with the introduction of new antigens, influences the maturation of the immune system, including the development of Tregs, which are crucial for maintaining immune tolerance, as demonstrated in murine studies [74, 75, 76].

The concept of “microbial age” in infants suggests that the microbiota evolves through specific stages, where certain microbial taxa act as markers of both microbiota development and indicators of potential immune maturation. Indeed, the presence of certain taxa can predict age [77]. A delay in the predicted microbial age is associated with diseases such as cystic fibrosis [78] and asthma [79]. Notably, microbial age can be positively influenced by growing up on a farm [79], an environment known to be protective against asthma [80, 81]. The concept of “microbial age” is specifically relevant to the gut microbiome, where gradual, diet‐driven changes mark microbial milestones, and the absence or delay of beneficial bacteria can lead to issues such as impaired immune development and increased disease susceptibility [82]. In contrast, the airway microbiome undergoes rapid, niche‐driven colonization, with the presence of pathogens such as *Moraxella* or *Haemophilus* being the primary concern [83]. Consequently, the milestone or “microbial age” concept does not apply to the airways, and understanding these distinctions is crucial when studying host–microbial interactions in the context of asthma.

## 3 Environmental, Respiratory, and Intestinal Microbiota and Their Impact on Asthma

### 3.1 Environmental Microbiota: From Farm Dust to Urban Air

Numerous epidemiological studies have shown that early‐life farm exposures have a protective effect against asthma, often referred to as the “farm effect” (Figure 1). Early research from Europe showed that children living on farms had lower allergy rates than their rural peers not raised on farms [84, 85]. These studies indicated that prolonged exposure to farm animals and consumption of cow's milk reduced allergy incidence [85]. A seminal study investigating the cause of the farm effect demonstrated an inverse relationship between endotoxin levels in bedding—indicative of microbial exposure—and the prevalence of atopic diseases among rural children [86]. Another study demonstrated that exposure to stables and consumption of unprocessed cow's milk independently provided protective effects against asthma and atopy [85]. A meta‐analysis confirmed the protective role of raw milk consumption in early childhood against asthma, even in non‐farm rural children, suggesting that the benefits of farm milk are independent of other farm exposures [87]. Notably, three different exposures—contact with cows, straw, and farm milk consumption—were independently found to replicate the farm effect on asthma in the GABRIELA cohort [88]. Recent findings from the PASTURE cohort indicate that exposure to animal sheds in early life significantly protects farm children from hay fever, with optimal protection requiring continuous farm milk consumption from infancy to school age [89]. Although both farm environmental exposures and raw milk consumption are likely to contribute to asthma protection, they may do so through different mechanisms.

The protective effect of farm environments is partly attributed to exposure to diverse microbial products, such as endotoxin, muramic acid, and β‐glucans [86, 90, 91]. A study from the GABRIELA cohort found that farm dust had significantly higher microbial diversity, which inversely correlated with asthma diagnosis [80]. A “microbial diversity score” explained a significant portion of the farm effect on asthma, with farm children's bedroom dust microflora resembling that in animal sheds in the same cohort [92]. In non‐farm homes, asthma risk decreased as the living‐room floor dust bacterial microbiota composition became more similar to that of farm homes [93]. Surprisingly, only a single pediatric cohort study has investigated a potential link between the farming environment and the gut microbiota so far [79]. This cohort from the PASTURE study demonstrated that fecal bacterial communities capable of producing SCFAs, particularly butyrate, contribute to asthma protection. Inverse associations were found between asthma, fecal butyrate levels, bacterial taxa predicting butyrate production (e.g., *Coprococcus* and *Roseburia*), and the abundance of the gene encoding butyryl‐coenzyme A (CoA):acetate‐CoA‐transferase, a key enzyme in butyrate metabolism. These findings suggest that microbiota may protect against asthma through the gut–ung axis (see Section 5.1). Additionally, an in vitro study showed that farm dust reduces IL‐33 expression in rhinovirus‐infected and TNF‐α/IFN‐γ–exposed airway epithelial cells, pointing to potential protective mechanisms [94].

Building on these groundbreaking European studies, researchers have utilized a unique real‐life case–control study to investigate the farm effect in a controlled manner. A few studies have investigated two distinct US farming populations: the Amish and the Hutterites. Despite their similar histories and genetic backgrounds, the Amish and Hutterites differ in farming practices: the Amish practice traditional single‐family dairy farming without modern technology, exposing children early to various farm animals, whereas the Hutterites live on large communal farms and utilize modern technology. These differences in lifestyle are reflected in the prevalence of asthma, which is significantly lower among the Amish (5.2%) compared to the Hutterites (21.3%) [95]. Amish children, exposed to protective farm environments, exhibit distinct immune cell profiles compared to Hutterite children. Specifically, peripheral blood leukocytes (PBLs) from Amish children show increased neutrophil proportions and decreased eosinophil proportions [81]. Gene expression profiling confirmed these differences, with 18 genes overexpressed in Amish PBLs clustering around innate immune genes, including TNF and IRF7, which are crucial for responding to microbial stimuli.

### 3.2 Respiratory Microbiota: Low in Numbers but Essential Gatekeepers

Although the respiratory tract harbors a relatively low biomass of microorganisms, particularly compared to sites such as the gut, emerging evidence suggests that the airway microbiota plays a crucial role in early immune development and is a key factor in the onset of respiratory conditions, including childhood asthma. This low microbial load, particularly in pediatric samples, necessitates rigorous methods for sampling and analysis, as contamination can obscure true microbial signals [96]. Despite these challenges, studies investigating the early‐life airway microbiota and its association with asthma have produced consistent findings, particularly compared to studies on the gut microbiota [55, 56, 97]. Specifically, multiple cohorts have demonstrated a strong link between the composition of the respiratory microbiota in the first months of life and the subsequent development of wheeze and asthma (Figure 1).

More than a decade ago, one of the pioneer studies from the COPSAC cohort, using culture‐based methods, found that colonization of the hypopharynx with *Streptococcus pneumoniae* , *Moraxella catarrhalis* , or *Haemophilus influenzae* at 1 month of age significantly increased the risk of recurrent wheeze, severe exacerbations, and hospitalization by Age 5 [55]. The study also showed that neonatally colonized children had higher blood eosinophil counts and total IgE levels, and a higher prevalence of asthma compared to non‐colonized children. Subsequent studies using culture‐independent techniques confirmed and expanded on these findings. Two Australian studies found that microbial profiles dominated by *Moraxella*, *Streptococcus*, and *Haemophilus* were significantly more frequent in children with acute respiratory infections (ARIs) compared to healthy controls [56, 57]. Although all three genera were linked to ARI, only early asymptomatic colonization by *Streptococcus*, detected in 14% of children at around 2 months of age, was significantly associated with chronic wheeze by Age 5 [56]. In a follow‐up study, the same authors identified slightly different results. They found that in early‐sensitized children, asymptomatic colonization of the upper airways by *Streptococcus*, *Haemophilus*, and *Moraxella* was associated with an increased risk of chronic wheeze by Age 5 [57]. However, in children who had not developed early allergic sensitization, colonization by these same bacteria was associated only with transient early wheeze, which resolved by the fourth year of life. In a large multicenter cohort of infants hospitalized for bronchiolitis, increased nasal *Moraxella* and *Streptococcus* species post‐hospitalization were associated with recurrent wheezing by Age 3 [98]. Of note, a few studies present contrasting findings, such as an increase in *Neisseria* abundance linked to wheeze by Age 2 [99], a *Staphylococcus*‐dominant profile in the first 6 months associated with recurrent wheeze and persistent asthma [100], and the presence of *Veillonella* and *Prevotella* at 1 month correlating with asthma by Age 6 in a COPSAC follow‐up study [97]. Given that these microbial changes occur early, well before disease onset, it can be hypothesized that they either contribute to immune dysregulation or arise as a consequence of a pre‐existing dysregulated microenvironment in the airways. In line with the observations made from early‐life studies, others have reported that both asthmatic children and adults exhibit an airway microbiota enriched with Proteobacteria, particularly *Moraxella* and *Haemophilus* species, compared to healthy controls [101, 102]. Mechanistically, *Moraxella catarrhalis* has been shown to trigger a strong inflammatory response, including neutrophil infiltration and elevated levels of TNF‐α and IL‐6, which could contribute to the exacerbation of allergic airway inflammation [103]. This infection can promote the expansion of Th17 cells, further aggravating inflammation, especially when bacterial exposure coincides with allergen sensitization. In contrast, commensal bacteria such as *Prevotella* spp. elicit weaker immune responses, potentially allowing their tolerance within the respiratory system [104]. These findings highlight that specific bacterial species may actively contribute to immune dysregulation, whereas others may support immune tolerance and influence asthma development.

Another key question in the field of childhood asthma is the transition from early‐life wheeze to persistent asthma, and whether changes in the airway microbiota could be contributing to this progression. Indeed, wheeze is one of the main clinical manifestations of asthma and commonly begins in early life, with up to 50% of children experiencing wheezing episodes before turning 6 [105]. A dysbiotic microbiota composition, such as *Moraxella* dominance and reduced diversity, has been linked to distinct wheeze phenotypes in preschoolers, specifically correlating with neutrophilic inflammation [106]. Although these symptoms are often temporary and resolve by the time children reach school age, about 30% of cases persist and develop into childhood asthma [107]. A multi‐omics study investigating the transition from preschool wheeze to asthma provided key insights into the distinct molecular signatures associated with this progression [108]. The first multi‐omic factor, combining different types of biological data to reveal complex molecular patterns, identified immune signatures involving neutrophil and Th1‐associated pathways, along with specific lipids and metabolites linked to inflammation in recurrent wheeze. The second factor revealed a trajectory toward Type 2‐high asthma, marked by airway epithelial gene expression changes, altered bacterial communities, and transcriptionally active pathogens like *Haemophilus influenzae* . These changes were detected even in the absence of acute symptoms, emphasizing the role of microbial activity and airway epithelial changes in driving the progression from wheeze to asthma.

### 3.3 Gut Microbiota: A Call for More Data

Over the last decade, numerous longitudinal studies have investigated the relationship between infant fecal microbiota and the future development of asthma or atopy. Initially, it was believed that higher microbial diversity was beneficial, as suggested by one of the early cohort studies linking lower alpha diversity in the first month to asthma at Age 7 [109]. However, while another study also noted decreased alpha diversity associated with future asthma [110], the vast majority [82, 111, 112, 113, 114, 115, 116] found no significant differences in diversity in children who developed asthma. The field has now shifted toward the idea that subtle changes in community composition, such as the lower abundance of certain beneficial microbes or microbial groups identified through differential abundance testing, may have a significant impact.

Many studies on gut microbiota and asthma derive from the Canadian CHILD cohort [82, 110, 111, 112], which can introduce a potential bias in the field, as findings are often based on similar or overlapping datasets. In a 2015 study, the authors identified a decrease in the fecal abundance of four bacterial genera at 3 months of age—*Faecalibacterium*, *Lachnospira*, *Rothia*, and *Veillonella*—which was associated with atopy and wheezing at 1 year [111]. In a follow‐up study examining preschool asthma outcomes at 4 years, it was found that the earlier findings held true only for one of these genera, *Lachnospira*, and additionally, an increased abundance of *Clostridium neonatale* species at 3 months was linked to asthma diagnosis at Age 4 [112]. A separate study from the same cohort using shotgun metagenomics found different species, such as lower abundance of *Anaerostipes hadrus*, *Fusicatenibacter saccharivorans*, *Eubacterium hallii* , and *Blautia wexlerae* life [82]. Another key study found that the highest risk group for asthma showed a lower relative abundance of *Bifidobacterium*, *Akkermansia*, and *Faecalibacterium* [48]. Interestingly, findings from an independent COPSAC cohort [114] did not show significant differences at early time points (1 week or 1 month), contrasting with the above‐mentioned studies that observed differences before 1 year [79, 111, 112, 113]. However, for those children born to asthmatic mothers within the COPSAC cohort who developed asthma by Age 5, the microbiota at 1 year showed decreased levels of *Faecalibacterium, Bifidobacterium*, *Roseburia*, *Alistipes*, *Lachnospiraceae*, *Ruminococcus*, and *Dialister*, with higher levels of *Veillonella* [114]. Notably, the *Veillonella* findings were opposite to those of the CHILD cohort, where lower levels of *Veillonella* were observed in babies who later developed asthma [114]. However, decreased relative abundance of *Bifidobacterium* aligned with findings from two previous studies [48, 113], whereas low *Faecalibacterium* was consistent with studies of the CHILD cohort [110, 111] alveolar type II (AT2) cells (Figure 1). More recent studies found lower levels of *Lachnospira*, as previously observed [111, 114], *Lachnobacterium*, *Lachnospira*, and *Dialister* [115], with later asthma, and a lower relative abundance of *Bacteroidetes* among other bacterial genera [79, 116]. Interestingly, studies conducted in children from rural Ecuador, a non‐industrialized setting, revealed strikingly different results, with higher levels of *Streptococcus*, *Veillonella*, and *Ruminococcus* observed at 3 months, linked to atopic wheeze at 5 years [113]. Altogether, these studies highlight the challenges in interpreting the results of these cohorts with only a subset of the observations occurring consistently across more than one study. The reasons for this are unclear but could be due to methodological, outcome (asthma, wheeze, and atopy), and geographical differences.

## 4 Timing of Early‐Life Exposures and Their Long‐Term Impact on Asthma Protection

### 4.1 Timing Is Key: The Critical Impact of When Microbial Exposure Occurs

The timing of microbial exposure is crucial in influencing its impact on immune development, with early‐life colonization directing immune maturation and maintaining mucosal homeostasis, a concept mainly supported by murine studies and emerging hints from human research. Disruptions during this time, due to factors such as antibiotic use, dietary changes, or birth delivery methods, can lead to dysregulated immune responses and increased susceptibility to asthma. Understanding this “window of opportunity” is essential for developing interventions that ensure optimal microbial exposures occur at the right time [117].

Several landmark murine studies have highlighted the importance of timing in microbial exposure by examining the effects of recolonizing mice at different time points or administering antibiotics at various stages of development. Germ‐free (GF) mice colonized with a conventional microbiota at birth for the first 9 weeks, but not in adulthood, exhibited reduced iNKT cell accumulation in the colon and lungs, thereby preventing airway hyperreactivity and inflammation and protecting adult mice from allergic asthma symptoms [118]. To further pinpoint the critical timing of microbial colonization, a study exposed GF mice to specific pathogen‐free (SPF) flora at six time points: Days 2, 7, 14, 21, 28, and 35. While adult mice cohoused with SPF mice did not show reduced high IgE levels, exposure at birth completely inhibited IgE induction. Colonization up to 1 week after weaning (Day 35) fully protected pups from elevated IgE in adulthood [119]. Others have demonstrated that additional microbial stimulation can be protective against asthma, as shown in a study where *Helicobacter pylori* infection in neonatal mice significantly reduced airway hyperresponsiveness and inflammation through the induction of Tregs [120]. This protective effect was most pronounced when the infection occurred early in life (6 days after birth), as adult infection did not provide the same benefits. Similarly, *Bifidobacterium longum* supplementation during the perinatal period (first 6 weeks), but not in adulthood, induced the expansion of Foxp3+ Tregss, providing protection against allergic inflammation [121]. In addition to recolonization and supplementation, several murine studies have demonstrated that early‐life antibiotic‐induced perturbation of the microbiota in newborn mice, but not adults, can significantly disrupt immune development and increase susceptibility to allergic airway inflammation [122, 123, 124, 125].

Although defining the precise early‐life window for host–microbial interactions in humans is challenging, we can draw conclusions by analyzing epidemiological data, for example, on antibiotic treatments and environmental exposures in relation to future asthma outcomes. In a meta‐analysis of 52 studies investigating the association between infant antibiotic exposure and the risk of childhood asthma, the strongest incidence of asthma was observed with antibiotic exposure during the first week of life, compared to exposures at later ages (6 months, 1 year, or 2 years). Additionally, an increased risk of asthma was noted as the number of antibiotic courses increased from 1 to 2 to 3–4, with the risk plateauing at five courses [126]. From an exposure perspective, children younger than 1 year who were exposed to stables and consumed farm milk demonstrated lower frequencies of asthma compared to those who were exposed to these factors between the ages of 1 and 5 years [85]. A follow‐up study from the CHILD cohort, which also included population‐based epidemiological data, found that the 26% decrease in asthma incidence among young children correlated with reduced antibiotic prescriptions during infancy [127]. Altogether, while murine models suggest early weeks are crucial, the field often refers to the first 100 days of life as significant for humans (Figure 1) [6].

### 4.2 Long‐Term Consequences: Lessons From Cohorts Follow‐Ups

As discussed, longitudinal studies have provided compelling evidence that the gut and lung microbiota, and their dysbiosis during a critical time window, are linked with the development of childhood asthma. However, these studies often focus on early disease outcomes, typically assessing children at ages 1 year [111], 4 years [48, 112], 5 years [127, 114] ), and up to 6 years [79]. Thus, a significant question remains: What are the long‐term consequences of these early‐life host–microbial perturbations? Understanding these implications is crucial, particularly since asthma remission during adolescence is common, with reported remission rates ranging from 16% to 60% [128].

The answer is not straightforward. Follow‐up studies from the CHILD cohort revealed that of the four bacterial genera initially identified at 3 months of age in relation to atopic wheeze at 1 year [111], only one genus, *Lachnospira*, was associated with an asthma diagnosis at Age 4 [112]. Additionally, another taxon, *Clostridium neonatale*, was linked to asthma at 4 years but not to atopic wheeze at 1 year in the same cohort. The COPSAC birth cohort stands out as perhaps the only cohort capable of addressing the long‐term consequences of early‐life microbial colonization on asthma, with follow‐ups conducted every 6 months from birth up to Age 7, and additional assessments at ages 12 and 18 [129]. This follow‐up study found that early colonization with *Streptococcus pneumoniae* , *Haemophilus influenzae* , and/or *Moraxella catarrhalis* (1 month of age) was associated with persistent wheeze/asthma during the first 7 years of life, with the difference in risk more pronounced at 4 years of age, attenuated at Age 6, and no longer evident at Age 12 or 18. Therefore, early‐life airway microbiota perturbations may be associated with a more transient, early‐onset form of asthma.

Interestingly, unlike the respiratory microbiota, the impact of environmental microbiota, such as farm exposure, appears to persist into adulthood. In keeping with this, a large Finnish cohort study demonstrated that early childhood contact with farm animals reduced the risk of asthma by 26% at Age 31 [130], although the impact of environmental microbiota was not directly investigated. Another extensive study found that this exposure provided lifelong protection against allergic rhinitis, which was also evident in elderly participants aged 61–65 [131]. Furthermore, recent findings from the PASTURE cohort revealed that exposure to animal sheds during the first 3 years of life significantly protected farm children from hay fever at 10 years [89]. However, it is noteworthy that this study identified the continuous consumption of farm milk from infancy through school age as necessary to achieve a protective effect. Altogether, these findings underscore the importance of long‐term follow‐ups and the integration of all microbiota—respiratory, gut, and environmental—to fully understand their impact.

## 5 Cross‐Organ Connections and Asthma

### 5.1 Established Connections: The Gut–Lung Axis

The gut–lung axis represents a critical pathway through which the gut microbiota influence respiratory health, particularly in the context of asthma [132]. Central to this interorgan crosstalk are metabolites produced by gut microbiota [8], such as SCFAs and secondary bile acids. The gut–lung axis has been primarily explored through murine studies, which offer insights into the mechanistic underpinnings of the gut–lung connection. A decade ago, several landmark studies elucidated key mechanisms through which dietary interventions and microbial interactions modulate immune responses and protect against airway inflammation. One prominent mechanism involves the role of SCFAs, which are metabolites produced through the fermentation of dietary fibers by gut bacteria. SCFAs, such as acetate and propionate, play significant roles in immune regulation. A high‐fiber diet enhances SCFA production, which has been shown to suppress allergic airway disease by promoting Tregs through the inhibition of histone deacetylase 9 (HDAC9) and the subsequent transcription of Foxp3 [133]. Another mechanism involves the influence of SCFAs on bone marrow hematopoiesis and DC function. Propionate, a specific SCFA, affects the maturation of DCs, leading to their high phagocytic capacity but reduced ability to stimulate Th2 effector functions in a GPR41‐dependent manner, thereby protecting against allergic airway inflammation [134]. Bile acids also play a significant role in the gut–lung axis, influencing both microbial composition in the gut and immune responses in the lung. Initially synthesized by the host as primary bile acids, they are further metabolized by gut microbiota into secondary bile acids such as deoxycholic acid and lithocholic acid [135]. These secondary bile acids possess antimicrobial properties, which allow them to modulate the gut microbiota by inhibiting certain microbial populations and promoting others [136, 137]. Ursodeoxycholic acid, a secondary bile acid modified by microbial activity, has been shown to reduce eosinophilic airway inflammation [138]. This reduction is achieved through the interaction with DC nuclear farnesoid X receptors, which play a role in modulating immune responses. Similarly, chenodeoxycholic acid, a primary bile acid, has been shown to alleviate allergic airway disease through its agonist activity on farnesoid X receptors in the lungs [139].

While Section 3.3 provided valuable insights into the taxonomic differences in early‐life gut microbiota among children cohorts in relation to asthma, shifting the focus to the functional aspects of these microbes, particularly their metabolites, is crucial, as it would allow insights to be drawn from the murine mechanistic studies. To date, most information about microbial metabolites from human studies relates to SCFAs. Lower fecal acetate levels at 3 months were linked to further development of atopy and wheeze in both the CHILD cohort [111] and the Ecuadorian infants study [113]. In addition, a study from the European PASTURE cohort found that higher levels of fecal butyrate and propionate were linked to a reduced incidence of atopy by Age 6 and showed a trend toward lowering asthma risk [140]. Lastly, urinary sulfated bile acids glycolithocholate, glycocholenate, and glycohyocholate were found to be increased in atopic wheezers of the CHILD cohort, whereas tauroursodeoxycholate was increased [111]. These findings highlight the growing importance of investigating microbial metabolites in human studies, as understanding their functional roles may bridge the gap between taxonomic shifts and mechanistic insights.

### 5.2 Emerging Concepts: The Skin–Lung Axis and the Gut–Brain–Lung Axis

The gut–lung axis is without a doubt the best‐documented interorgan connection that links early life microbiota to asthma development. However, the reality is more complex, as we are part of a multi‐organ system where microbial metabolites from the gut can reach virtually all distant tissues, including the bone marrow, brain, and adipose tissue, as demonstrated by isotope‐labeled bacteria administered to GF mice [141]. This systemic distribution is rapid and pervasive; for instance, oral administration of isotope‐labeled butyrate reaches the skin within 45 min [142]. In addition to gut microbiota metabolites reaching the skin, the skin microbiota itself might influence the lungs through allergen penetration and systemic immune responses. Recent experimental evidence indicates that immune maturation of the skin, influenced by age and the microbiome, plays a critical role in controlling the nature and severity of allergic diseases in both the skin and lungs [9]. Neonatal skin shows impaired chemokine and alarmin production and has fewer skin‐resident antigen‐presenting cells (APCs) compared to adults. The chemokine profile and APC populations in naive skin from neonatal SPF mice and adult GF mice are lower than those in adult SPF mice. Cohousing GF mice with SPF mice restored chemokine expression and APC seeding in the skin, indicating that microbial colonization is crucial for these processes.

Another cross‐organ axis that has gained significant attention in recent years is the gut–brain axis. A dysregulated gut microbiome can negatively affect innate immune cells in the central nervous system, glial cells, by compromising the integrity of both the intestinal and blood–brain barriers, potentially contributing to neurodegenerative diseases [143]. Additionally, recent evidence suggests that the lung microbiota might also influence brain function, indicating a potential lung–brain axis [144]. Local microbiota changes caused by intratracheal antibiotics increased lipopolysaccharide (LPS)‐producing bacteria, which crossed the blood–brain barrier and triggered a protective Type I interferon response in microglia, improving conditions such as experimental autoimmune encephalomyelitis [145]. Could there be a link between gut microbiota, the nervous system, and the development of childhood asthma? Although direct evidence for such a link is lacking, recent findings suggest intriguing associations between early‐life gut microbiota, neurotransmitter production, and Treg‐mediated immune tolerance [10]. The study identified gut microbiota as the primary source of serotonin in the neonatal gut, surpassing epithelial cells, and noted that these microbes downregulated monoamine oxidase A to reduce serotonin breakdown. Gut microbes isolated from human neonatal stool, such as *Staphylococcus aureus* , *Clostridium perfringens* , and *Klebsiella grimontii*, were also capable of producing serotonin. This serotonin signaled directly to T cells, increasing intracellular indole‐3‐acetaldehyde and inhibiting mTOR activation, which promoted the differentiation of Tregs. Oral administration of serotonin to neonatal mice led to long‐term, T cell–mediated, antigen‐specific immune tolerance toward both dietary antigens and commensal bacteria. Consequently, it is reasonable to speculate that such priming could influence asthma development, given that Tregs are associated with asthma protection and severity later in life.

## 6 Beyond Bacteria: Emerging Roles of Fungi and Helminths

### 6.1 Fungi: Debating Their Contribution to Asthma Development

The human microbiota encompasses a diverse array of microorganisms, including viruses, fungi, archaea, helminths, and bacteria, with bacteria being the most prevalent. However, the fungal component, known as the mycobiota, is often overlooked and referred to as the “forgotten friend” [146]. In recent years, there has been a growing interest in the role of fungi, particularly in relation to atopy and asthma [11]. Despite this increased attention, fungi remain significantly less abundant than bacteria, constituting less than 0.1% of the total gut microbial metagenome [147, 148, 149] and are scarcely detected in shotgun metagenomics from airway samples (unpublished data). In a study designed to explore the distinct roles of the bacterial and fungal components of the microbiota, GF mice were recolonized with bacterial consortia, fungal consortia associated with asthma, or both [150]. The findings revealed that recolonization with bacteria, but not fungi, induced physiological changes, such as reduced small intestinal length and increased cecal weight. Beyond these physiological effects, bacterial colonization significantly influenced the metabolome, whereas fungi did not. Notably, bacterial colonization, whether alone or in combination with fungi, reduced airway inflammation, whereas fungi alone had no effect. However, when present with bacteria, fungi promoted macrophage and monocyte infiltration in the lungs.

In healthy airways, the presence of fungi appears to be largely influenced by environmental exposure, rather than the establishment of a stable fungal community. Indeed, a significant portion of fungal taxa in bronchoalveolar lavage (BAL) samples are shared with the home environment, with around 75% overlap, indicating that many fungi in the airways are transient and inhaled from the surroundings [151]. Additionally, the composition of the airway mycobiome changes with the seasons, with only *Malassezia restricta* consistently detected over time, further supporting the transient nature of these fungi [152]. A study from the Breathing Together cohort found that nasal samples from neonates were rich in environmental fungi [61]. Furthermore, multi‐omics data integration showed significant associations between host immune gene expression and bacteria, but not with fungi, suggesting that fungi in the airways are primarily a result of environmental influx rather than forming a stable microbial community, and they may have little to no impact on the host immune system under steady‐state conditions. While environmental fungi may not pose a problem for healthy airways, atopy, particularly to mold allergens, is closely associated with childhood asthma severity [153, 154]. Interestingly, children exposed to higher levels of spores and pollen during the first 3 months of life, as measured by ambient spore and pollen air concentration, have an increased risk of early wheezing, potentially explaining the variation in childhood asthma risk based on birth month [155].

With increasing evidence of the airway bacterial microbiota's role in wheeze and asthma, and rising interest in fungi, a few studies have examined the airway mycobiota in both children and adults with asthma. In a small cohort of asthmatic children, BAL samples revealed a higher abundance of fungi such as *Rhodosporidium*, *Pneumocystis*, *Leucosporidium*, and *Rhodotorula* compared to non‐asthmatic children, without changes in fungi typically linked to sensitization, such as *Aspergillus* and *Alternaria* [156]. No differences in fungal diversity were observed. In a study of asthmatic adults and controls, *Trichoderma*, *Alternaria*, *Cladosporium*, and some *Fusarium* species were more abundant in asthmatics, whereas *Blumeria, Mycosphaerella*, and other *Fusarium* species were decreased [157]. Notably, *Trichoderma* showed increased relative abundance in patients with a Th2 high profile when compared to low Th2. Furthermore, fungal diversity was lower in endotracheal brush samples from asthmatics, although this reduction was not replicated in BAL samples. A recent study on children with asthma found that a higher abundance of *Malassezia globosa* in nasal samples was linked to fewer annual Yellow Zone (YZ) episodes, which are early signs of loss of asthma control, and a reduced risk of these episodes progressing to severe asthma exacerbations [158]. Another study aiming to unravel the roles of environmental and respiratory mycobiota in relation to Th2 endotypes found that during exacerbations, patients with severe asthma had a respiratory mycobiome more similar to the indoor mycobiome than during stable periods [159]. Differences in indoor fungal communities were observed between asthma phenotypes, but not in sputum samples. Altogether, the lack of consistent findings across studies suggests that the mycobiota may not play a significant role in asthma, whereas environmental fungi could exacerbate the condition in sensitized patients.

What about the presence of fungi in the gut? Recent research has started to address this question in children at risk of developing atopy and asthma. One of the first studies in the field identified three groups of participants based on their neonatal intestinal bacterial communities, each with different risks for developing atopy by Age 2 and asthma by Age 4 [48]. The group at highest risk showed an increased relative abundance of fungal genera such as *Candida* and *Rhodotorula*, along with a decrease in *Malassezia*. In another study conducted in rural Ecuador (ECUAVIDA), infants who developed atopic wheeze by Age 5 exhibited a higher fungal load and increased abundance of *Pichia kudriavzevii* [113]. Notably, fungal dysbiosis was more strongly linked to the onset of atopic wheeze than bacterial dysbiosis. In an attempt to model these findings in experimental animals, neonatal mice were exposed to *Pichia kudriavzevii*, leading to increased inflammatory responses during allergic airway disease later in life and linked to elevated Th2 and Th17 inflammation responses [160]. Additionally, when *Pichia kudriavzevii* was cultured with physiologically relevant concentrations of SCFAs, such as acetate, butyrate, and propionate, its growth was inhibited. Although the experimental approach was rudimentary, this suggests that bacterial SCFAs could potentially inhibit fungal growth, providing some insights into the bacteria–fungal interactions in the gut. A recent study from the PASTURE cohort found that, unlike bacteria, the estimated fungal age did not correlate with asthma, although the presence of the fungal genus *Alternaria* at 2 months was linked to subsequent bacterial maturation [79]. Altogether, there is even less consensus among studies on the role of fungi in atopy and asthma compared to bacteria. However, unlike the airways where fungal presence is mainly environmentally driven, the gut hosts a more stable fungal community that interacts complexly with bacteria, potentially influencing disease outcomes.

### 6.2 Helminths: Modulators of Immune Responses in Asthma

Helminths have co‐evolved with humans throughout history and exhibit an extraordinary ability to modulate the host immune response. Although billions of people living in developing areas still suffer from the morbidity that these pathogens can incur, efforts to control infectious disease through improved hygiene have led to decreased infections in developed countries [161]. Interestingly, the absence of intestinal helminths has been postulated to underlie the increasing prevalence of allergic and autoimmune diseases within westernized societies [162], leading to speculation that the eradication of helminths contributes to the epidemiological observations underlying the hygiene hypothesis. Indeed, studies in people have shown an inverse relationship between atopy [163]. Yet human studies investigating multiple aspects of allergy have proved inconclusive, other than providing evidence that *Ascaris lumbricoides* is positively associated with bronchial hyperreactivity in children and atopy in adults [164]. *A. lumbricoides* migrates through the lungs as part of its lifecycle [165], and tropomyosins from *A. lumbricoides* can cross‐react with homologous antigens present in common allergens such as HDM and cockroach [166, 167].

In contrast to the human data, animal models have provided clear evidence that helminth infection can protect against experimental allergic airway inflammation [162]. The means by which helminths exert their regulatory role in animal models include the promotion of Tregs [168] and the secretion of molecules that interfere with host pathways involved in the allergic response, such as eicosanoid production [169] and IL‐33 signaling [170]. Of note, infection with the natural murine intestinal helminth, *Heligmosomoides polygyrus*, altered the intestinal microbiota, resulting in increased availability of SCFAs, which attenuated allergic inflammation through a mechanism involving the host SCFA receptor GPR41 and increased Treg responses [171]. This study additionally showed that modulation of allergic asthma by the helminth‐altered microbiota could be transferred to uninfected animals through fecal transfer. That helminths alter the intestinal microbiota is now well established, with helminth–microbiota interactions now reported to impact several inflammatory diseases, including allergy, arthritis, colitis, and obesity [172].

## 7 Conclusion

This review has highlighted the role of early‐life microbial communities in shaping childhood asthma, with the gut and respiratory microbiota receiving the most attention. The gut microbiota, particularly through the gut–lung axis, plays a central role in immune regulation, with metabolites such as SCFAs promoting immune tolerance and reducing inflammation. Disruptions in gut microbial composition during early life, especially involving species like *Faecalibacterium* and *Bifidobacterium*, have been linked to future asthma development and atopic wheeze. The respiratory microbiota, although lower in biomass, could also play a role, with early colonization by *Haemophilus*, *Streptococcus*, and *Moraxella* associated with increased wheezing and asthma risk in children. The timing of these microbial exposures is critical, as early disruptions can have long‐lasting effects on immune development. In humans, this critical window is believed to fall within the first 100 days of life.

Despite progress, key questions remain, particularly whether microbial dysbiosis is a cause or consequence of asthma. Integrating multi‐omics data (from both host and microbes) and using advanced computational models will help identify patterns across microbial communities that isolated datasets cannot reveal. Addressing these challenges requires collaboration across immunology, microbiology, and data science. Ultimately, large‐scale data from diverse cohorts could enable the development of predictive models and personalized treatments, improving childhood asthma management. From an intervention perspective, early‐life probiotic supplementation for asthma prevention has often been unsuccessful, likely because it focuses on only a few microbes (e.g., *Lactobacilli*) rather than those specifically altered in children with asthma. Given the inconsistencies in identifying which bacterial taxa are beneficial and the limited success of probiotic studies, direct supplementation with SCFAs may offer a more effective approach.

## Conflicts of Interest

The authors declare no conflicts of interest.

## Data Availability

Data sharing not applicable to this article as no datasets were generated or analysed during the current study.
